# Assessing individual learning styles of undergraduate medical students utilizing Kolb's learning style inventory

**DOI:** 10.12669/pjms.41.8.11890

**Published:** 2025-08

**Authors:** Nusrat Zareen, Farah Rashid, Tabassum Alvi, Rida Fatima Sajid

**Affiliations:** 1 Nusrat Zareen, Department of Anatomy, Watim Medical & Dental College, Rawalpindi, Pakistan; 2 Farah Rashid, Department of Community Medicine, NUST School of Health Sciences, National University of Sciences and Technology (NUST), H-12 Sector, Islamabad 44000, Pakistan; 3 Tabassum Alvi, Department of Psychiatry, Avicenna Medical College, Lahore, Pakistan; 4 Rida Fatima Sajid, Department of Anatomy, Army Medical College, NUMS, Rawalpindi, Pakistan

**Keywords:** Abstract Conceptualization, Clinical students, Experiential Learning Theory, Problem solving, Undergrad students

## Abstract

**Background &Objective:**

David A. Kolb, an educational theorist, proposes that learning is a process, and that experience plays a key role in how individuals learn. In his Experiential Learning Theory, he has introduced a Learning Style Inventory, as a tool to assess and categorize individuals based on their preferred ways of learning. Our objective was to assess the individual learning styles of undergraduate medical students based on Kolb’s learning style inventory (KLSI).

**Methods:**

In this cross sectional observational study a total of 142 medical students of 1^st^, 2^nd^ and 3^rd^ year MBBS, of a private Medical College, Rawalpindi were included through convenient sampling. After ethical approval study was conducted from March – August 2024. Kolb’s inventory was converted to google form and disseminated to students after, informed consent and allocating secret code to all. Responses received, were retrieved as excel sheets and also entered to SPSS software version 26. Individual students’ responses were plotted on Excel spread sheet graphs to attain the kite shapes, determining the learning style of each student. The study duration was from March - December 2024.

**Results:**

The highest learning style amongst students was observed to be converging. However, when analyzed via chi square test, no significant difference was observed either between the students of the three years or between the learning styles of pre and post clinical students. There was transitory pattern of learning style preference among the 02 pre-clinical years of 1^st^ and 2^nd^ years.

**Conclusion:**

It was concluded that learners exhibit different learning style preferences, that if known to the learner and facilitators would be helpful in the process.

## INTRODUCTION

Students’ learning styles may be defined as different ways in which they understand, collect information, concentrate, process and recollect facts within academic domain.^1^ This helps to access how students observe information, relate to it and react to learning environment.^2^ Knowing and understanding the students learning pattern has a focus for improving academic performance since long. Kolb’s Learning Style Inventory (KLSI) is one of the most authoritative frameworks categorizing learners based on how they process and perceive information.^3^ This model, developed in 1984, identifies four distinct learning styles Diverging, Assimilating, Converging, and Accommodating each derived from the interplay between two dimensions: how learners perceive (Concrete Experience vs. Abstract Conceptualization) and how they process (Active Experimentation vs. Reflective Observation) information. Students integrate with different types of knowledge and information according to their learning styles.^3^

Recently the theorist Kolb has reemphasized his learning styles quoting Diverging learners to observe and gather information, showing strength in viewing situations from multiple perspectives; Assimilating learners to favor logical analysis and theoretical models; Converging learners excel in applying ideas to practical problems; and Accommodating learners rely on hands-on experiences and adaptability.^4^ Recognizing these preferences is valuable in developing learner-centered teaching approaches, especially in clinical and problem-based settings where diverse cognitive styles influence performance and engagement

Health education is a multifaceted field where various perspectives intersect. Due to its fast evolving demands it is crucial to reconsider the foundational principles of higher education, shifting the focus from traditional teaching to the broader teaching-learning process.^5^ There are many reports which consider that self-awareness is very effective in any improvement in ones learning process.^6^ However, some researchers are convinced that Learning styles are basically wrong concept with not much benefits on the learning process.^7^

As pointed out by Muniyapillai et al. (2023)^8^ different teaching strategies such as demonstration, small group discussion, self presentation, and laboratory work, are interpreted as different learning styles and had different impact on the students learning. Researchers have grouped these modes into different learning preferences, and instructors must know and use the best of these to facilitate students learn.^9^,^10^

Recent educational research emphasizes upon the concept of self-regulated learning and meta-cognition. Empowering students to understand their own learning styles and adjust their behaviors accordingly while learning is a key aspect of improving the quality of education. It is emphasized that assisting students to know themselves and to operate in a meta-cognitive fashion is vital for the quality of the learning process.^11^ Therefore, a study was designed to analyze and help undergrad medical students understand and further implement their own learning style to their educational training. Also, the results were expected to help the facilitator better design their teaching strategies.

Despite the widespread application of Kolb’s Experiential Learning Theory (ELT) in educational and professional development settings, significant research gaps remain, particularly concerning its effectiveness across diverse cultural and digital learning environments. Kolb’s model, emphasizes a cyclical process of concrete experience, reflective observation, abstract conceptualization, and active experimentation, assuming that learners benefit most when teaching aligns with their preferred learning style. However, empirical support for this matching hypothesis remains inconsistent, and critics argue that learning styles may not be as fixed or predictive as the theory suggests. Moreover, the theory was conceptualized primarily in Western contexts, raising concerns about its applicability in non-Western, collectivist cultures where different educational values and learner behaviors prevail. Additionally, the rise of virtual and hybrid learning modalities presents new challenges for experiential learning, particularly in replicating “concrete experiences” and real-time reflection. There is also a dearth of longitudinal studies assessing whether aligning teaching strategies with Kolb’s learning styles leads to improved academic or clinical outcomes in health professions education. These limitations suggest the need for updated research that validates ELT within multicultural, technologically mediated, and healthcare-specific learning environments (Haukedal et al., 2022).^12^

In an attempt to fill up the above gap, current study was designed to assess the individual learning styles of undergraduate medical students based on Kolb’s learning style inventory.

## METHOD

This cross-sectional observational study was conducted on undergraduate 1^st^, 2^nd^ & 3^rd^ year medical students of Watim Medical and dental college, Rawalpindi (WMCR). The study was conducted for a period of 06 months, from March – August 2024.

### Ethical Approval:

It was obtained from the Research Ethics Committee WMCR #WMCR/R&D(ERB)/2024/117 dated 12/03/24.

**Fig.1:**
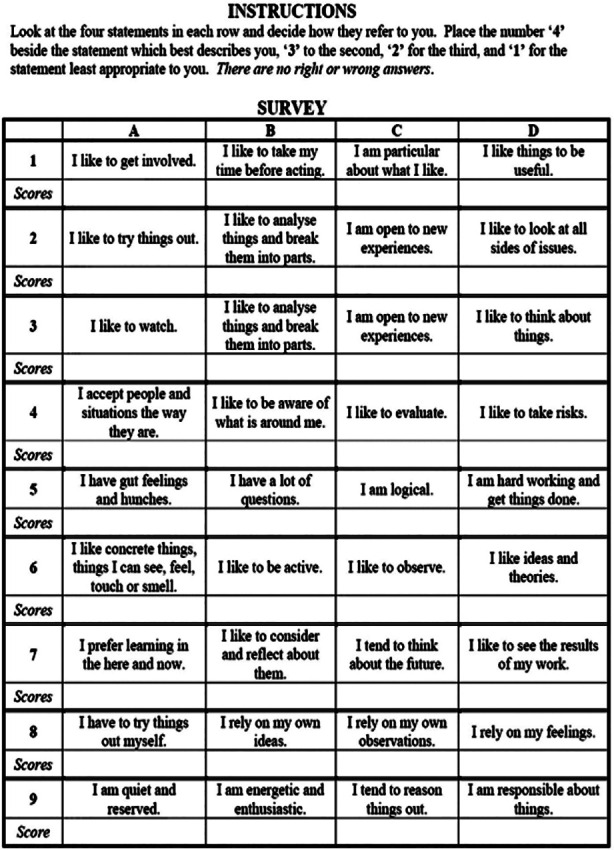
Kolb learning style inventory KLSI administered to students.^16^

Kolb Learning Style Inventory questionnaire^13^ (Fig.1) was converted to Google form and shared over WhatsApp group of students to record the data. This study employed a convenient sampling method to gather initial insights into the learning styles of medical students. The identity of students was kept confidential, and all the participants were allocated a code. A broad inclusion was intentionally applied, and all enrolled willing MBBS students of 1^st^ - 3^rd^ year MBBS were eligible to participate. No stringent exclusion criteria were applied (except the wrongly filled out forms) to maximize participation and reflect the diversity of learning approaches across the student body.

After screening the correctly filled forms, a total of 142 students were included in the study. Data obtained from the questionnaire was retrieved over Excel spread sheets and further recorded on SPSS software. Individual responses to all the domains assessed in the inventory were digitally plotted over Excel sheet, obtaining a “kite shape” based on the Kolb’s experiential learning theory. The “kite” diagram was used to represent the learning styles as a visual tool to display the relative strengths of an individual’s preferences in each of the four learning modes. Chi square test was applied on the qualitative data and respective learning styles of undergrad students were statistically compared.

### Plotting the kite:

**Fig.2:**
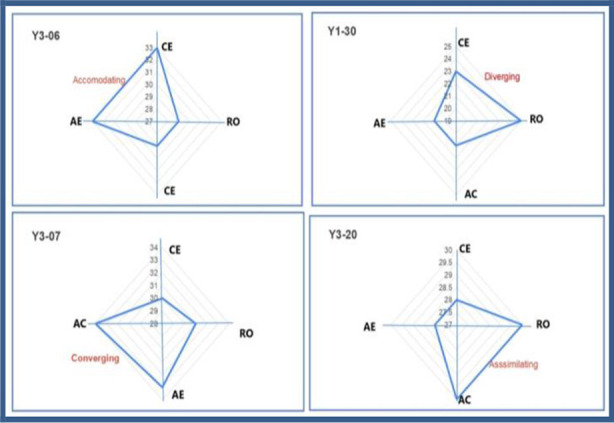
The kite shape “visual tool” for interpreting the students learning preferences. **CE:** Concrete experience, **RO:** Reflective observation, **AC:** Abstract conceptualization, **AE:** Active Experimentation

For every participant student, the sum of individual preferences for each modality of the four learning stages, namely Concrete experience, (CE), Reflective observation (RO), Abstract conceptualization (AC) and Active Experimentation (AE) were calculated obtained from the kLSI. The respective aggregate score of the student for each style was Digitally plotted on the excel sheets. Plotting relative distribution of scores among the four domains shaped the “kite”. The inclination of the ‘Kites’ were interpreted, learning style was further categorized into Accommodator, Divergers, Assimilator or Convergers based on Kolb’s theory (Fig.2).

## RESULTS

**Fig.3:**
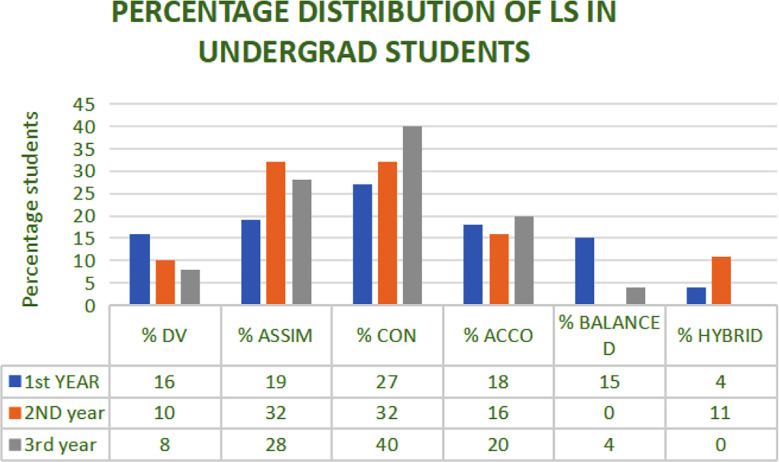
Percentage students expressing Kolb’s learning style (KLSI)

All the participants responded to the questionnaire according to their own preferences. The data was collected for different tendencies, plotted as kites, and interpreted as their individual learning style namely; Divergers, assimilators, convergers or, accommodators. The participant students were 73 from 1^st^ year, 44 for 2^nd^ year and 25 from 3^rd^ year, summing up to a total of 142. Recorded percentage learners category from each academic year is summarized in (Fig.3)

Highest percentage of students were convergers (27%) in first year, a balance of assimilators & convergers (32% each) in 2^nd^ year and also convergers (40%) in 3^rd^ year.

The qualitative data was analyzed through Chi square test to compare the learning preferences, for all the three years (Table-I) and also for pre-clinical (1^st^ & 2^nd^ years) and clinical students of 3^rd^ years MBBS (Table-II), No significant difference was recorded among any of the groups. The predominant learning style adopted by both Pre clinical and clinical students was convergers, (Table-II).

**Table-I:** Comparison of learning style preferences of undergrad students

Personality type	Year of study			Total	P-value
	1st year	2nd year	3rd Year	
Divergent	12	4	2	18	0.07
Assimilator	14	14	7	35	
Convergr	20	14	10	44	
Accommodator	13	7	5	25	
Balanced	11	0	1	12	
Hybrid	3	5	0	8	
Total	73	44	25	142	

**Table-II:** Comparison of learning style preferences between clinical and pre-clinical students

Groups	Learners’ type						Total	P value
	Divergent	Assimilator	Converger	Accommodator	Balanced	Hybrid		
Preclinical	16	28	34	20	11	8	117	0.553
Clinical	2	7	10	5	1	0	25	
Total	18	35	44	25	12	8	142	

## DISCUSSION

For some time, there had been a trend of educational research on learning styles originally within the field of psychology. Khanda and Basnat recently have endorsed that knowing one’s own learning styles has positive impact on academic performance.^14^ Some studies also emphasize that learners need to be well aware of their learning styles preferences in order to maximize their academic output.^15^ In recent years, researchers believe that instructors must also know and use the best strategies to assist students to learn.^14^

The Medical college, where current study was conducted is in the process of transition from traditional to integrated curriculum. At the time of this study, new 1^st^ year class was inducted in modular integrated system, while the higher 2^nd^ and 3^rd^ year classes were following the traditional system.

This study embarks on exploration of individual learning styles of the students in all these three years identifying the distinct learning preferences based on four defined cognitive styles - Converging, Diverging, Assimilating, and Accommodating.^16^ Students with accommodating learning style are active participants in their learning, Assimilators are more indulged towards prepared exercises, Divergers prefer prepared lectures and convergers prefers interactive sessions.^17^

In our study the highest percentage of students from all three academic years preferred converging style followed by the assimilators. There was no statistically significant difference among pre clinical and clinical students, when compared through chi-square test. (Table-II)

However, according to previous data quoted in studies a more purposeful approach for pre-clinical medical years would have been Assimilators and Divergers. These personality types are more likely to thrive due to their preference for abstract concepts, theoretical knowledge, and reflection on learning. These learners benefit from lectures, textbooks, and PBL sessions. While for clinical students Convergers and Accommodators are better suited to the practical, hands-on nature of clinical training, where experiential learning through patient interaction and real-world problem-solving plays a central role.^18^

Our findings therefore, point out a gap in our induction system and also a lack of direction in our youth while choosing careers. However, it also highlights the potential in our students to grasp and evolve according to the demand of the profession, and the effort of the teachers to mold the potential in the correct learning direction. The pre clinical 1^st^ year students were majority of convergers instead of being assimilators or divergers, as an ideal group. While another pre-clinical class, the 2^nd^ year MBBS were recorded to be accommodators and finally the preliminary Clinical class of 3^rd^ year students were recorded to be the ideal Convergers, getting ready for more experiential learning through patient interaction.

As explained by educationalist, individuals with a converging learning style tend to excel in applying theoretical concepts to practical situations. They possess strong problem-solving and decision-making abilities, focusing on finding effective solutions to challenges. These individuals typically prefer working on technical tasks and solving practical problems. In structured learning environments, they are inclined to experiment with new ideas, engage in simulations, and participate in hands-on activities such as laboratory experiments and practical applications.^19^ All these qualities complement the clinical students very much, while interacting with real-life cases and endering best patient care.

The Experiential Theory further elaborates that learners move through all learning quadrants in different learning situations and with passage of time and experience adapt to one or two styles 20 it considers the stages and learning styles as indivisible, i.e., to achieve the styles, it is obligatory to merge the stages that can start in any of their phases, transit and combine them, thus forming a style.^21^ In our study the frequency of students adopting different learning styles in real time, is different for different academic years, yet with no statistically significant difference. However different individual styles in every class embarks the evolving learning patterns based on the personality types.

The hybrid learners observed in our study (Fig.3) correspond to the Kolb’s concept of experiential learners. According to this theory, learners tend to develop predisposition for certain learning styles based on their individual experiences, but they also alter over time depending on the context and the type of learning situation. This aligns with his idea that learners shift across different modes based on experience.^22^

The idea of “balanced learners” (Fig.3) can be explained as the interplay of the four learning styles. Each individual typically has a dominant style, but the theory also suggests that learners can develop and utilize all four styles to varying degrees over time.^23^ Balanced learners could be seen as those who are flexible in using different learning styles depending on the context, drawing from each of the four modes when needed.

Although KLSI has provided a useful way to understand student preferences, some research suggest that focusing too heavily on matching teaching to learning styles may not always lead to improved academic outcomes. Pashler et al.^24^ pointed out that learning styles are preferences rather than abilities, and overemphasizing them, can limit flexibility. Kolb himself stressed the importance of developing learning flexibilitym^25^,^26^ which means engaging in all modes of learning. In higher education, where students face complex, multidisciplinary challenges, relying solely on one learning style may not be the most effective approach. Rather, adapting and integrating different learning modes can help students become more versatile learners. Meanwhile teachers’ information of students learning preferences may also be very helpful towards the learning process.^27^

The observed variability, of undergraduate learning styles and conflicting opinions of educationists, emphasizes over more longitudinal studies on the subject.

### Limitations

The small and unequal number of participants from respective classes was a limitation to the study. Also, the students found the Inventory difficult to fill in, and many forms had to be discarded. A more demonstrative instruction for better compliance is suggested for future studies.

## CONCLUSION

Kolb’s Learning Style Inventory offered a valuable tool for understanding individual learning preferences in higher education, promoting self-awareness and helping students align their study strategies. The information is not only beneficial for learners but also a good guide for facilitators to plan their teaching strategies according to the learning style of the learners.

Different learners exhibit different learning styles preferences, that if known to the learner and or facilitators would be helpful in the processes. However, the authors do believe that Higher education demands a complex, integrative approach to problem-solving and learning, and an inflexible adherence to a specific learning style may hinder academic growth in diverse disciplines. Hence the hybrid strategy might be a better approach towards learning. The importance of career counseling has also come to light in this study to pace up with the modern concepts of experiential learning.

### Authors’ Contribution:

**NZ:** Study design, drafted the manuscript, Statistical analysis of the data and responsible for integrity of research.

**FR:** Study conceptualization, Critical review, final approval of the study.

**TA:** Critical revision for intellect content. Literature search, critical review.

**RFS:** Literature search, IT support. Critical analysis.

## References

[1] Adu Ko, Pylman N, Adu Eo (2020). Learning Styles as Correlates of Grade 6 Learner's Mathematics Performance In Buffalo City Municipality In South Africa. e-Bangi.

[2] Yaman A, Bredeche N, Caylak O, Leibo JZ, Lee SW (2022). Meta-control of social learning strategies. PLoS Comput Biol.

[3] Koohestani HR, Baghcheghi N (2020). A comparison of learning styles of undergraduate health-care professional students at the beginning, middle, and end of the educational course over a 4-year study period (2015–2018). J Educ Health Promot.

[4] Kolb AY, Kolb DA (2021). The Experiential Educator:Principles and Practices of Experiential Learning.

[5] Sell K, Hommes F, Fischer F, Arnold L (2022). Multi-, Inter-, and Transdisciplinarity within the Public Health Workforce:A Scoping Review to Assess Definitions and Applications of Concepts. Int J Environ Res Public Health.

[6] Nancekivell SE, Sun X, Gelman SA, Shah P (2021). A Slippery Myth:How Learning Style Beliefs Shape Reasoning about Multimodal Instruction and Related Scientific Evidence. Cogn Sci.

[7] Newton PM, Miah M (2017). Evidence-Based Higher Education - Is the Learning Styles 'Myth'Important?. Front Psychol.

[8] Muniyapillai T, Kulothungan K, Abdul Malik SR, Jeevaraj SJ, Ashokan S, Ravichandran S (2023). Learning styles and their relationship with preferred teaching methodologies and academic achievement among medical students in teaching medical college, Tamil Nadu. J Educ Health Promot.

[9] Asharini DA, Hanafiah H, Kusuma GP (2023). Development of Classroom Management Based on Student Learning Style Database.

[10] Aldhaheri N, Al-Rahmi WM, Yahaya N, Kamin YB, Omar A (2023). Clustering Students Based on Gamification User Types and Learning Styles.

[11] Panadero E (2017). A Review of Self-regulated Learning:Six Models and Four Directions for Research. Front Psychol.

[12] Haukedal D, McCallum K, Davies M (2022). Reassessing learning style theories:a critical review of Kolb's model. Innov Educ Teach Int.

[13] Engels, Paul T (2010). Learning styles of medical students, general surgery residents, and general surgeons:implications for surgical education. BMC Med Educ.

[14] Khadka A, Basnet A, Jaiswal R, Karki S, Magar SS (2024). Learning styles, approaches and academic performance of second and third-year medical students of a medical college of Kathmandu:a descriptive cross-sectional study. Ann Med Surg (Lond).

[15] Wilkinson T, Boohan M, Stevenson M (2014). Does learning style influence academic performance in different forms of assessment?. J Anat.

[16] Ybek Özdemir D, Akalin A (2024). Mimetic teaching strategy in design education:relationship between students'learning style and creativity. J Design Plan Aesthet Res.

[17] Hydrie MZI, Naqvi SMZH, Alam SN, Jafry SIA (2021). Kolb's Learning Style Inventory 4.0 and its association with traditional and problem-based learning teaching methodologies in medical students. Pak J Med Sci.

[18] Liu J, Zhan J, Guo S (2021). Application of Kolb's experiential learning theory in medical education:A systematic review. J Med Educ.

[19] Sánchez AJ, D'Amico L (2023). A new framework for the visualization of Kolb's learning styles:a focus on the application in higher education. J Educ Res Pract.

[20] Figueiredo LDF, Silva NCD, Prado MLD (2022). Primary care nurses'learning styles in the light of David Kolb. Rev Bras Enferm.

[21] Karns GL (2020). Kolb's experiential learning theory and its application in the learning environment. J Educ Learn.

[22] Gibson C, Hall R, Sheehan M (2021). Hybrid learning in post-secondary education:The case for flexible and adaptive learners. J High Educ.

[23] López MA, Blázquez D (2020). Adaptation of learning styles in the classroom:A multidimensional approach to experiential learning. Int J Educ Res.

[24] Pashler H, McDaniel M, Rohrer D, Bjork R (2009). Learning styles:Concepts and evidence. Psychol Sci Public Interest.

[25] Torrance H, Thomas G (2021). Learning styles and Kolb's experiential learning theory:An exploration of flexibility in educational contexts. Educ Psychol Rev.

[26] Hung LY, Wang SM, Yeh TK (2023). Kolb's experiential learning theory and marine debris education:Effects of different stages on learning. Mar Pollut Bull.

[27] Haik Y (2014). Compatibility of Teaching Styles with Learning Styles:A Case Study. Eur J Educ Sci.

